# Psychometric properties of the Polish version of the 36-item WHODAS 2.0 in patients with hip and knee osteoarthritis

**DOI:** 10.1007/s11136-021-02806-4

**Published:** 2021-03-15

**Authors:** Agnieszka Bejer, Agnieszka Ćwirlej-Sozańska, Agnieszka Wiśniowska-Szurlej, Anna Wilmowska-Pietruszyńska, Renata Spalek, Alessandro de Sire, Bernard Sozański

**Affiliations:** 1 Institute of Health Sciences, College of Medical Sciences of the University of Rzeszow, University of Rzeszow, Rejtana Street 16C, 35-959 Rzeszow, Poland; 2 Faculty of Medicine, Lazarski University, Świeradowska Street 43, 02-662 Warsaw, Poland; 3 Rehabilitation Unit, Mons. L. Novarese Hospital, Str. Sotto Cerca, 13040 Vercelli, Moncrivello Italy; 4 Department of Geriatrics, Neurosciences, Orthopedics, Center for Geriatric Medicine (CEMI), Institute of Internal Medicine and Geriatrics, Catholic University of the Sacred Heart, L.go F.Vito, 8, 00168 Rome, Italy; 5 Physical and Rehabilitative Medicine, Department of Health Sciences, University of Eastern Piedmont, Viale Piazza D’Armi 1, 28100 Novara, Italy

**Keywords:** 36-item WHODAS 2.0, International Classification of Functioning, Disability and Health, Psychometrics, Knee Osteoarthritis, Hip Osteoarthritis

## Abstract

**Purpose:**

To examine psychometric properties of the Polish version of the 36-item WHO Disability Assessment Schedule 2.0 (WHODAS 2.0) in the population with hip and knee osteoarthritis (OA).

**Methods:**

This was a longitudinal study with repeated measures during retest examinations. Subjects from a Polish Specialist Hospital (age = 68.3 ± 9.2years, 71% female, 44.2% knee OA, 55.8% hip OA) were tested three times. They completed the Polish version of the 36-item WHODAS 2.0, the SF-36 Health Survey 2.0, the Western Ontario and Macmaster Universities Osteoarthritis Index 3.1, the Hospital Anxiety and Depression Scale, and the Numerical Rating Scale.

**Results:**

The 36-item WHODAS 2.0—Polish version demonstrated high internal consistency (Cronbach’s alpha for total = 0.94), and test–retest reliability (Total ICC_2,1_ = 0.98). High construct validity was found as 12 out of 15 a priori hypotheses (80%) were confirmed. Most domains and Total Scores in the 36-item WHODAS 2.0 (Total ES = − 0.62, SMR = − 1.09) showed a moderate degree of responsiveness. Minimal clinically important difference (MCID) for the Total WHODAS 2.0 was 3.29 in patients undergoing rehabilitation for knee or hip OA.

**Conclusions:**

The Polish version of the 36-item WHODAS 2.0 assesses disability according to ICF in a reliable, valid and responsive way. Therefore, it provides considerable support in clinical practice and national and international scientific research of patients with hip or knee OA.

## Plain English summary

Osteoarthritis (OA) is a very common cause of pain, stiffness and disability worldwide. The World Health Organization has recently developed the 36-item WHO Disability Assessment Schedule (WHODAS 2.0) as a questionnaire aimed to assess disability status on the basis of the conceptual framework contained in the International Classification of Functioning, Disability and Health (ICF). Therefore, we sought to evaluate whether the Polish version of the WHODAS 2.0 might be used to assess health and disability in the Polish population with hip and knee OA.

The 36-item WHODAS 2.0 is available in English. It was translated into Polish and culturally adapted. However, before using it among Polish patients, it should be checked whether the Polish version is equivalent to the original. We investigated whether the Polish version of the 36-item WHODAS 2.0 assesses health and disability in patients with hip and knee OA appropriately.

Taken together, our findings indicate that the Polish version of the 36-item WHODAS 2.0 might be used by clinicians and researchers in Poland. The questionnaire showed to be useful in better understanding the subjective opinion of patients, with hip and knee OA, about their health condition and the limitations affecting them in everyday life due to the disease. Apart from clinical tests and imaging, information obtained with the 36-item WHODAS 2.0 allows the most accurate and comprehensive adjustments to the required treatment of patient's, monitoring their effectiveness, and making modifications according to the ICF model.

## Introduction

Osteoarthritis (OA) is a very common cause of pain, stiffness and disability worldwide, affecting 303 million people globally in 2017 [1, 2]. Osteoarthritis of the lower extremities results in a significant restriction of mobility, causing the sufferer to have difficulties when walking and performing routine daily activities [3]. Additionally, pain associated with this disease significantly reduces the patient's physical activity, leading to further adverse changes in the body [4]. Osteoarthritis also has a negative impact on person’s mental well-being and on their quality of life, at the same time, it consumes a meaningful amount of health care resources and funds [2, 5]. Functional limitations caused by OA should be detected as early as is possible, in order to diagnose and treat the age-related degenerative progression [6]. Such treatments as glucocorticoid and hyaluronic acid intra-articular injections [7], physical therapies [8], and oxygen-ozone therapy [9], and physical exercise can be used to reduce the pain and improve the patient’s quality of life [10, 11].

Apart from objective methods, patient-reported outcome measures (PROMs) are recommended to determine an effective treatment program, this includes rehabilitation and monitoring its effects [12]. The PROMs were divided into condition- or disease-specific and generic measures. The first group showed greater potential to better differentiate groups by clinically salient symptoms and responsiveness to changes in the condition of the subjects [13]. Researchers and clinicians in Poland can use the available Polish language versions of the questionnaires to assess the health status of people with OA—the Western Ontario and Macmaster Universities Osteoarthritis Index (WOMAC) [14, 15], the Hip Disability and Osteoarthritis Outcome Score (HOOS) [16], the Knee Injury and Osteoarthritis Outcome Score (KOOS) [17], and the Knee Outcome Survey Activities of Daily Living Scale (KOS-ADLS) [18]. The generic PROMs available in the Polish version include the SF-36 Health Survey 2.0 (SF-36 v.2.0), which have been widely used in the assessment of quality of life [19, 20]. However, it follows a health-related quality of life model that is beginning to be replaced by more inclusive multidimensional models.

The generic PROMs is also the 36-item WHO Disability Assessment Schedule (the 36-item WHODAS 2.0). It was created by the World Health Organization (WHO) and was developed on the basis of the conceptual framework contained in the International Classification of Functioning, Disability and Health (ICF) [21, 22]. The ICF provides a description of the situation concerning human functioning and its limitations. It is a conceptual framework that adequately describes disability in people affected by OA [23]. In this scenario, a multicentre cross-sectional study [24], involving 864 patients, who were referred to thirteen Italian University outpatient clinics, showed that knee and hip OA were the most common pathological conditions (11.8%), and the most common altered ICF item was represented by “b280: sensation of pain” (76.3%).

The 36-item WHODAS 2.0, which is based on the ICF, differs from other research tools and might be applied in different cultures, general population or in clinical practice worldwide. Whilst determining the level of functioning, all disorders are treated equally, therefore this tool allows us to compare disabilities caused by different diseases. The 36-item WHODAS 2.0 also facilitates the design of health and health-related interventions, including rehabilitation, and the monitoring of their effectiveness [21, 22, 25, 26]. Extensive research on a population of *N* = 1565, in 14 countries showed that WHODAS 2.0 demonstrated strong clinimetric properties. These include the psychometric characteristics for internal consistency (Total Cronbach`s alpha is 0.98, for domains from 0.94 to 0.96), reliability (ICC is 0.98, for domains from 0.93 to 0.96), validity (the assumptions about the correlation of WHODAS 2.0 domains with reference questionnaires such as: London Handicap Scale, Functional Independence Measure, SF-36, SF-12, WHOQOL-100 and WHOQOL-BREF, where confirmed; validity factor analysis showed a close relationship between the factor structure position and domains, and between domains and the general factor of disability), and responsiveness (ES ranges from 0.46 for subjects with depression to 1.38 for people with schizophrenia) [22].

Federici et al. in an international systematic review from 2017 indicated that the 36-item WHODAS 2.0 was translated into 47 languages and dialects and used in 27 areas of research [27]. The questionnaire was translated and evaluated in patients with knee OA in Turkey [28], and amongst patients with various musculoskeletal problems, e.g. in Portugal [29], Germany [30] or Norway [31]. Garin et al. validated the 36-item WHODAS 2.0 amongst 1119 patients from 7 European Centers with 13 chronic conditions, including 297 people with OA [32].

Amongst the generic measures with proven psychometric properties, for assessing adult disability, available in Poland is the 12-item WHODAS 2.0 questionnaire [33]. It is recommended by the WHO to use for brief assessments in situations where time constraints do not allow for application of the longer version or where there is a need to use a short tool to study a large population [22]. The 36-item WHODAS 2.0 was validated in an elderly population [34] and in a group of people over 50 and patients with low back pain [35]. However, to date, the Polish version of the WHODAS 2.0 including 36 items has not yet been evaluated in OA patients in Poland.

Therefore, we sought to examine the psychometric properties of the Polish version of the 36-item WHODAS 2.0 in patients affected by hip and knee OA, to provide findings which would be useful for better evaluating health and disability.

## Methods

### **Study design and study population**

This was a longitudinal study with repeated measures during retest examinations. Participants were recruited from amongst consecutively admitted patients diagnosed with hip and knee OA from June 2019 to March 2020 at the rehabilitation ward of the holy family specialist hospital, Rudna Mała, Poland. They were included, provided they met the following inclusion criteria: (a) age ≥ 50 years, (b) suffering from hip or knee OA for at least 3 months, (c) native speaker of Polish, (d) informed and written consent were required to participate in the study. Exclusion criteria were: (a) coexisting neurological disorders, (b) other than OA, diseases or injuries located in any part of the lower limb that may induce different symptoms or/and disturb their function.

### Measures

#### The 36-item WHO Disability Assessment Schedule (WHODAS 2.0)

In accordance with WHO regulations the 36-item WHODAS 2.0 was translated and culturally adapted by the ICF Council at the Poland Health Protection IT Systems, led by Professor Anna Wilmowska–Pietruszyńska, based on the agreement with the WHO [34, 36]. A questionnaire was used to measure general disability and disabilities in six domains: cognition (DoC, 6 items), mobility (DoM, 5 items), self-care (DoSC, 4 items), getting along with people (DoGA, 5 items), life activities (DoLA, 8 items), participation (DoP, 8 items). Answers were classified according to a 5-point scale identifying the level of difficulty or problem (1 none; 2 mild; 3 moderate; 4 severe; 5 extreme or cannot do). The obtained results were converted according to the instructions, to a scale from 0 to 100, where 0 means no disability, whilst 100% means extreme disability [21, 22].

#### The SF-36 Health Survey 2.0 (SF-36 2.0)

It is a generic PROM, which consists of 36 items measuring eight domains: physical functioning (DoPF), role limitation related to physical problems (DoRLPP), bodily pain (DoBP), general health (DoGH), mental health (DoMH), vitality (DoV), social functioning (DoSF) and role limitation related to emotional problems (DoRLEP). In addition, the first four domains constitute physical component scale (PCS), whilst the next four—mental component scale (MCS). The answers provided by the respondents are normalized so that the score calculated on the basis, of the said answers is within the range of 0–100 pts, where the value 0 means the worst quality of life and the score of 100 pts is the best possible [17, 18]. (License agreement No.: QM030224).

#### The Western Ontario and Macmaster Universities Osteoarthritis Index 3.1 (WOMAC 3.1)

The questionnaire consists of 24 items that cover three subscales: pain, stiffness and physical function. These data are standardized to a range of values from 0 to 100 on a percentage scale, where 0 represents the worst health status whilst 100—the best health status. It was used to assess the functional status of patients [14, 15].

#### The Hospital Anxiety and Depression Scale (HADS)

It is a 14 part multiple choice questionnaire which measures the presence of symptoms of both anxiety (HADS-A) and depression (HADS-D). The final score for each subscale ranges between 0 and 21, where 0 represents the best health status and 21 the worst [37, 38].

#### The Numeric Rating scale (NRS)

The 11-point NRS was used, where 0 represents no pain at all, whilst 10 stands for the worst pain imaginable. The respondent was instructed to identify one number between 0 and 10, which was best representative of their pain intensity [39].

### **Study procedure**

Convenience sampling method was applied in our research. All consecutively admitted patients suffering from hip or knee OA from June 2019 to March 2020 meeting the inclusion criteria qualified for the study. The study was conducted by properly prepared and trained physiotherapists. The investigation was carried out using a one to one, pen and paper interview method. The participants were evaluated three times. The baseline examination (on admission to the rehabilitation ward; test 1) consisted of completing the Polish versions of all previously mentioned questionnaires. During the second examination (test 2; 2 days after test 1), patients completed only the 36-item WHODAS v. 2.0. Then the participants completed the 36-item WHODAS 2.0, and the NRS 4 weeks after completing a 21-days in-hospital rehabilitation program (test 3).

### **Sample size**

Post hoc analysis of the test effectiveness was conducted using the ICC with the null hypothesis ICC = 0.7, with a sample group size of 123 people. The estimated ICC value for the Polish population is 0.05. The accuracy of the test is extremely high, showing over 0.999 for each groups score total. This showed that the sample group size was satisfactory.

### **Statistical analysis**

The statistical analyses was conducted using R software version 3.6.2 [40]. The level of statistical significance was assumed at *p* ≤ 0.05. A normal distribution was determined using the Shapiro–Wilk test.

The sample and questionnaires applied were characterized using descriptive statistics (mean, standard deviation, range, frequencies). The Mann–Whitney *U* test for numerical data was used to comparison between groups from test 1 and 3, and for qualitative data the Pearson chi^2^ test. Floor effect was defined if more than 15% obtained the lowest possible score, ceiling effect occurred if more than 15% obtained highest possible score [41].

### Reliability analysis

#### Internal consistency

Internal consistency was verified with Cronbach`s alpha coefficient (α). A coefficient between 0.70 and 0.95 was considered as satisfactory [41, 42].

#### Reliability (test–retest)

The intra class correlation (ICC_2,1_), with a 95% confidence interval (CI) was used to assess the test–retest reliability. We assumed positive rating for reliability when the ICC amounted to ≥ 0.70 [41, 42].

#### Measurement error (test–retest)

The standard error of measurement (SEM) and minimal detectable change at the 95% level (MDC_95_) were used to assess error [43, 44].

### Construct validity analysis

#### Tested hypotheses

A priori hypotheses were formulated in line with the aim (assessment of the construct validity) and include expected relationships between the 36-WHODAS 2.0 and the comparison instruments (depending on the similarity of the construct), and the expected direction and magnitude of the correlation. The Pearson's correlation coefficient (PCC) was calculated. The indications for PCC *r* strength for validity were ≤ 0.30 = low, 0.3 ˂ *r* ˂ 0.6 = moderate and ≥ 0.60 = high [45]. These hypotheses also concern the ability of the questionnaire to differentiate between patients with various health status. Student's *t* test was used to assess differences in scores between ‘known groups’. Patients were divided according to the WOMAC 3.1 score: group with small and medium functional limitations (*N* = 62, 0–50 points) and group with big and very big functional limitations (*N* = 67, 51–100 points) and according to the NRS score: group with no pain or mild pain (*N* = 50, 0–4) and group with moderate and severe pain (*N* = 79, 5–10). Hypotheses were formulated by authors AB and AĆS independently, then overall agreement of the expected correlation were checked. Fifteen ones were chosen for the analysis (Table 1). If fewer than 25% of the hypotheses were rejected, construct validity of the 36-item WHODAS 2.0 was considered high, and for moderate validity 25–50% and for low validity more than 50% should be rejected [41].

**Table 1:** A priori hypotheses for assessment the of the construct validity of the 36-item WHODAS 2.0

No	A priori hypotheses
1	Correlation between the WHODAS 2.0 (Total) and the SF-36 2.0 (PCS) should be stronger than in between the WHODAS 2.0 (Total) and the SF-36 2.0 (MCS)
2/3	The WHODAS 2.0 (DoC) should correlate moderately, negatively with the SF-36 (MCS) and moderately, positively the HADS
4/5	The WHODAS 2.0 (DoM) should correlate highly, negatively with the SF-36 (DoPF) and the WOMAC
6/7	The WHODAS 2.0 (DoSC) should correlate highly, negatively with the SF-36 2.0 (DoPF) and the WOMAC
8/9	The WHODAS 2.0 (DoGA) should correlate highly, negatively with the SF-36 2.0 (DoSF) and moderately, negatively with the SF-36 (MCS)
10/11	The WHODAS 2.0 (DoLA) should correlate highly, negatively with the SF-36 2.0 (DoRLPP and PCS)
12/13	The WHODAS 2.0 (DoP) should correlate highly, negatively with the SF-36 2.0 (DoSF) and moderately, negatively with the SF-36 (DoGH)
14/15	The WHODAS 2.0 (Total) has sufficient discriminatory power. Differentiates between patients in different functional states according to the WOMAC (group 0–50 pts vs. 51–100 pts) and with different levels of pain in the NRS (0–5 vs. 6–10)

*WHODAS 2.0* the 36-item World Health Organization Disability Assessment Schedule 2.0, *SF-36 2.0* the SF-36 Health survey 2.0, *The HADS* the Hospital Anxiety and Depression Scale, *WOMAC 3.1* the Western Ontario and Macmaster Universities Osteoarthritis Index 3.1

### Responsiveness

#### The standard effect size (ES) and standardized response mean (SRM)

ES is defined as a score change in the 36-item WHODAS 2.0 (between test 1 and test 3) divided by baseline SD, and the SRM was calculated by dividing the mean score change by the SD of that score change. Absolute values of 0.20 or less, 0.21–0.79, and 0.80 or greater represent small, moderate, and large responsiveness, respectively, for ES and SRM [46].

#### The minimal clinically important difference (MCID) with its standard error (SE)

The MCID was calculated using the anchor method. NRS was used as an anchor and a change by 1 point on the NRS was considered a "minimally detectible" one. Linear regression analysis was used to find the amount of change in WHODAS 2.0 (between test 1 and test 3) that was associated with the change by 1 point on the NRS [47].

## Results

According to the inclusion and exclusion criteria, in test 1 the resultant sample group of participants, was *N* = 129, i.e. 81% of the patients treated at the rehabilitation ward of the holy family specialist hospital in Rudna Mała, between June 2019 and March 2020, with knee or hip OA. (The mean age ± standard deviation was 68.3 ± 9.2 years, range 51–88, 71% were female, 44.2% of whom were diagnosed with knee OA and 55.8% with hip OA).

123 people participated in test 2 (5 patients refused to participate in test 2, 1 patient was transferred to another ward), and 98 people participated in test 3 (24 patients refused to participate in test 3, 1 patient died). The general socio-demographic and clinical characteristics are presented in Table 2.

**Table 2:** General socio-demographic and clinical characteristics of the study population (Total sample *N* = 129, and Responsiveness sample *N* = 98)

Variables	*N* = 129, test 1	%		*N* = 98, test 3	%	*t*	*p*
Age (years)	68.28 ± 9.18			66.46 ± 11.00		0.65	0.516
Gender							
Female	92	71.3		77	78.6	1.54	0.215
Male	37	28.7		21	21.4		
Diagnosis							
Knee osteoarthritis	57	44.2		48	49.0	0.51	0.473
Hip osteoarthritis	72	55.8		50	51.0		
Place of residence							
Countryside	73	56.6		54	55.1	0.05	0.975
Town (below 20,000 residents)	28	21.7		22	22.5		
City	28	21.7		22	22.5		
Education							
Primary education	29	22.5	23		23.5	0.49	0.922
Vocational education	25	19.4	20		20.4		
Secondary education	41	31.8	27		27.6		
Higher education	34	26.4	28		28.6		

Age is expressed as mean ± standard deviation

*N* number, *%* percentage, *t* U Mann–Whitney test/chi^2^ Pearson test

The PROMs absolute values, floor and ceiling scores from test 1 are presented in Table 3.

**Table 3:** Absolute values, floor and ceiling scores of all questionnaires (*N* = 129)

Questionnaire	Domain	$\overline{x}$ ± SD	Range	Floor score (%)	Ceiling score (%)
WHODAS 2.0	Cognition	14.88 ± 18.74	0–70	39.53	0.00
	Mobility	57.07 ± 25.96	0–100	1.55	3.10
	Self-care	32.09 ± 21.82	0–100	11.63	0.78
	Getting along	30.68 ± 22.73	0–91.67	18.60	0.00
	Life activities	53.33 ± 30.68	0–100	8.53	11.63
	Participation	45.54 ± 18.23	0–100	1.55	0.78
	Total score	38.33 ± 17.28	1.09–81.52	0.00	0.00
SF-36 2.0	Physical functioning	33.37 ± 20.16	0–85	0.78	0.00
	Role physical	38.18 ± 25.51	0–100	13.95	3.88
	Body pain	39.05 ± 22.09	0–100	5.43	3.88
	General health	44.73 ± 17.47	0–95	1.55	0.00
	Vitality	50.35 ± 16.64	10–90	0.00	0.00
	Social functioning	61.14 ± 26.25	0–100	0.78	21.71
	Role emotional	66.02 ± 28.21	0–100	3.10	28.68
	Mental health	57.95 ± 15.82	12–88	0.00	0.00
	PCS	37.53 ± 16.4	7.14–86.43	0.00	0.00
	MCS	57.97 ± 15.98	15.71–91.43	0.00	0.00
HADS	Anxiety	7.09 ± 3.6	0–21	1.55	0.78
	Depression	6.04 ± 4.04	0–20	3.88	0.00
WOMAC 3.1	WOMAC	54.3 ± 16.58	12–95	0.00	0.00
NRS	NRS	5.05 ± 1.92	2–10	0.00	1.55

*WHODAS 2.0* the 36-item World Health Organization Disability Assessment Schedule 2.0, *SF-36 2.0* the SF-36 Health Survey 2.0, *HADS* the Hospital Anxiety and Depression Scale, *WOMAC 3.1* the Western Ontario and Macmaster Universities Osteoarthritis Index, *NRS* the Numerical Rating Scale, $\overline{x}$ mean, *SD* standard deviation, *%* percent

The floor and ceiling effect for the total score of the 36-item WHODAS 2.0 was not present. However, over 15% of the respondents reported the lowest possible score for the domains: DoC (39.53%) and DoGA (18.60%) (Table 3).

### Reliability analysis

#### Internal consistency

The 36-item WHODAS 2.0 internal consistency was satisfactory with α range from 0.77 for DoP and DoSC to 0.95 for DoLA and 0.94 for total score (*N* = 129) (Table 4).

**Table 4:** Results of the reliability analysis: internal consistency (*N* = 129), test–retest reliability and measurement error (*N* = 123)

WHODAS 2.0	Cronbach's alpha (*α*)	ICC_2,1_(95% CI)	SEM	MDC
Cognition	0.859	0.971 (0.961–0.979)	3.12	8.65
Mobility	0.886	0.949 (0.931–0.962)	6.03	16.71
Self-care	0.773	0.95 (0.933–0.963)	4.81	13.33
Getting along	0.852	0.97 (0.96–0.978)	3.98	11.03
Life activities	0.952	0.974 (0.965–0.98)	4.81	13.33
Participation	0.767	0.922 (0.897–0.942)	4.88	13.53
Total score	0.943	0.981 (0.974–0.986)	2.34	6.49

*WHODAS 2.0* the 36-item World Health Organization Disability Assessment Schedule 2.0, *ICC*_*2,1*_ intra class correlation, *CI c*onfidence interval, *SEM* standard error of measurement, *MDC* minimal detectable change

#### Reliability and measurement error (test–retest)

The value of ICC_2,1_ (*N* = 123) for the 36-item WHODAS 2.0 was very high, it ranged from 0.92 for DoP to 0.97 for DoLA and 0.98 for total score. SEM ranged from 3.12 from DoC to 6.03 for DoM and 2.34 for total score. MDC ranged from 8.65 for DoC to 16.71 for DoM, and 6.49 for total score (Table 4).

### Construct validity

#### Hypotheses testing

Table 5 shows the construct validity using the PCC for the Polish version of the 36-item WHODAS 2.0 and the reference questionnaires. As hypothesized, scores that represent the same areas correlated strongly, indicating that both questionnaires measure a similar construct. At the same time, scores that represent less convergent regions correlate moderately or weakly, depending on the similarity of the construct. A priori formulated hypotheses that have been confirmed are marked in bold and underlined.

**Table 5:** Construct validity as measured by Pearson’s correlation (*r*) for the 36-item WHODAS 2.0 v. chosen domains of the SF-36 2.0, the HADS, and the WOMAC 3.1 (*N* = 129)

Questionnaire	WHODAS 2.0						
	Cognition	Mobility	Self-care	Getting along	Life activities	Participation	Total score
SF-36 2.0							
Physical functioning	− 0.442	− **0.776**	− **0.69**	− 0.74	− 0.595	− 0.539	− 0.792
Role physical	− 0.358	− 0.503	− 0.574	− 0.638	− **0.549**	− 0.553	− 0.662
Body pain	− 0.319	− 0.544	− 0.487	− 0.456	− 0.492	− 0.357	− 0.555
General health	− 0.449	− 0.463	− 0.483	− 0.439	− 0.495	− **0.394**	− 0.572
Vitality	− 0.502	− 0.391	− 0.54	− 0.477	− 0.4	− 0.539	− 0.602
Social functioning	− 0.358	− 0.371	− 0.356	− **0.362**	− 0.306	− **0.389**	− 0.458
Role emotional	− 0.442	− 0.283	− 0.405	− 0.383	− 0.279	− 0.496	− 0.473
Mental health	− 0.482	− 0.282	− 0.481	− 0.412	− 0.283	− 0.501	− 0.517
PCS	− 0.52	− 0.791	− 0.759	− 0.792	− **0.699**	− 0.625	− **0.876**
MCS	− **0.571**	− 0.41	− 0.567	− **0.48**	− 0.396	− 0.616	− **0.548**
HADS							
Anxiety	**0.409**	0.313	0.417	0.404	0.281	0.422	0.475
Depression	**0.54**	0.358	0.428	0.443	0.33	0.424	0.536
WOMAC 3.1	− 0.458	− **0.728**	− **0.708**	− 0.599	− 0.62	− 0.521	− 0.761

A priori formulated hypotheses that have been confirmed are marked in bold and underlined

A priori formulated hypotheses that have been rejected are marked in bold

All correlations had *p* ≤ 0.001

*WHODAS 2.0* the 36-item World Health Organization Disability Assessment Schedule 2.0, *SF-36 2.0* the SF-36 Health Survey 2.0, *The HADS* the Hospital Anxiety and Depression Scale, *WOMAC 3.1* the Western Ontario and Macmaster Universities Osteoarthritis Index 3.1

*r* Pearson's correlation coefficient, *L* = *r *≤ 0.3, *M* = 0.3 < *r < *0.6, H = *r* ≥ 0.6

A priori formulated three hypotheses that have been rejected are marked in bold. No strong correlations were found between the domains of the Polish version of 36-item WHODAS 2.0—DoGA, DoLA, DoP and the reference domain of the SF-36 2.0: DoSF. DoRLPP.

The 36-item WHODAS 2.0 shows appropriate discriminatory power, i.e. there is a statistically significant difference between people with different functional status according to the WOMAC 3.1 and with different intensity of pain according to the NRS (Table 6).

**Table 6:** Construct validity of the 36-item WHODAS 2.0 as measured by comparison between ‘known groups’ (*N* = 129)

WHODAS 2.0		WOMAC 3.1		*p*	NRS		*p*
		0–50	51–10		0–4	5–10	
Cognition	$\overline{x}$ ± SD	21.29 ± 20.9	8.96 ± 14.26	*p* < 0.001*	9.8 ± 17.23	18.1 ± 19.05	*p* = 0.014*
Mobility	$\overline{x}$ ± SD	71.88 ± 16.7	43.38 ± 25.56	*p* < 0.001*	47 ± 28.52	63.45 ± 22.11	*p* < 0.001*
Self-care	$\overline{x}$ ± SD	42.74 ± 19.93	22.24 ± 18.73	*p* < 0.001*	24.8 ± 23.41	36.71 ± 19.53	*p* = 0.002*
Getting along	$\overline{x}$ ± SD	39.11 ± 21.95	22.89 ± 20.69	*p* < 0.001*	21.33 ± 20	36.6 ± 22.46	*p* < 0.001*
Life activities	$\overline{x}$ ± SD	66.13 ± 29.61	41.49 ± 26.81	*p* < 0.001*	44.2 ± 29.14	59.11 ± 30.39	*p* = 0.007*
Participation	$\overline{x}$ ± SD	52.55 ± 16.43	39.05 ± 17.5	*p* < 0.001*	39.83 ± 16.97	49.16 ± 18.18	*p* = 0.004*
Total score	$\overline{x}$ ± SD	47.77 ± 14.38	29.59 ± 15.06	*p* < 0.001*	30.98 ± 16.8	42.98 ± 16.01	*p* < 0.001*

$\overline{x}$ mean, *SD* standard deviation, *p* student's *t* test, *WHODAS 2.0* the 36-item World Health Organization Disability Assessment Schedule 2.0, *WOMAC 3.1* the Western Ontario and Macmaster Universities Osteoarthritis Index 3.1, *NRS* the Numerical Rating Scale

*Statistically significant (*p* ≤ 0.05)

Twelve out of 15 a priori assumed hypotheses (80%) were confirmed. This indicates high construct validity of the 36-item WHODAS 2.0.

### Responsiveness

#### Standard effect size (ES) and standardized response mean (SRM)

There was a significant change in all domains of the 36-item WHODAS 2.0 and the total scores between test 1 and test 3 (*N* = 98). All the results decreased significantly, thus a significant reduction in the degree of disability of the patients was achieved. We calculated also ES and SRM for the 36-item WHODAS 2.0. Apart from DoC (for ES = − 0.13), all other domains and Total score showed a moderate degree of responsiveness, respectively, as signified by ES and SRM values (Table 7).

**Table 7:** Results of the responsiveness analysis (*N* = 98)

WHODAS 2.0	Change test 3 versus test 1			*p*	ES	SRM	MCID	SE
	$\overline{x}$	Me	SD					
Cognition	− 2.45	0.00	9.56	*p* = 0.015*	− 0.13	− 0.26	0.89	0.32
Mobility	− 16.33	− 12.50	19.39	*p* < 0.001*	− 0.63	− 0.84	5.15	0.69
Self-care	− 9.29	− 10.00	14.73	*p* < 0.001*	− 0.43	− 0.63	2.55	0.53
Getting along	− 9.52	− 8.33	11.54	*p* < 0.001*	− 0.42	− 0.82	2.52	0.44
Life activities	− 9.90	− 10.00	20.43	*p* < 0.001*	− 0.32	− 0.48	2.83	0.72
Participation	− 8.55	− 8.33	13.60	*p* < 0.001*	− 0.47	− 0.63	2.55	0.48
Total score	− 10.79	− 9.78	9.89	*p* < 0.001*	− 0.62	− 1.09	3.29	0.37

*WHODAS 2.0* the 36-item World Health Organization Disability Assessment Schedule 2.0, $\overline{x}$ mean, *Me* median, *SD* standard deviation,* p* paired student's *t* test, *ES* effect size, *SRM* standardized response mean, *MCID* minimal clinically important difference, *SE* standard error

*Statistically significant (*p* ≤ 0.05)

#### Minimal clinically important difference (MCID) and standard error (SE)

The largest MCID was found in the case of DoM (5.15 ± 0.69), whilst the smallest was found in the case of DoC (0.89 ± 0.32). The MCID for total scores was 3.29 ± 0.37 (*N* = 98) (Table 7).

## Discussion

Considering the biopsychosocial disability model of ICF, assessment of activity limitations and restrictions in participation should be a part of the comprehensive assessment of patients with knee and hip OA. The 36-item WHODAS 2.0 is a standardized tool designed to measure health and disability and is used in clinical practice and research [34]. To the best of our knowledge, we conducted the first assessment of the psychometric properties of the Polish version of the 36-item WHODAS 2.0 in patients with knee and hip OA. Most of the proposed hypothesis described in the methodology were proven. The results of our study provide support for high reliability, validity, and responsiveness of the Polish version of the questionnaire that can be used to assess disability amongst patients with OA.

The internal consistency of the Polish version of 36-item WHODAS 2.0 was assessed using α and was found to be 0.94 (ranged 0.77–0.95). This confirms very good internal consistency, because scores lower than 0.70 may indicate no correlation between items on the scale, whilst higher than 0.95 may indicate item redundancy [41]. Kutlay et al. studied 225 patients with knee OA in Turkey, and Silva et al. validated the Portuguese version amongst 204 patients with musculoskeletal pain, obtaining similarly results in terms of internal consistency for the Total score, respectively α = 0.93 and α = 0.84 [28, 29]. The results of the studies by other authors also indicate a very good internal consistency of this questionnaire [30, 32, 48–51].

According to Terwee et al. positive ratings for test–retest reliability may be given when the ICC is ≥ 0.70 [41], so good repeatability of the Polish version of 36-item WHODAS 2.0 was proven for the group of patients with knee and hip OA (0.98 for total, ranged 0.92–0.97). These findings are consistent with another report regarding musculoskeletal disorders, which confirm good repeatability of the various language versions of the questionnaire. Baron et al. analyzing data obtained from 172 patients with early inflammatory arthritis and obtained the ICCs value of 0.94 for total (ranged 0.82–0.96) [50]. Similarly, Kutlay et al. obtained the ICC value of 0.97 for total, and Silva et al. received 0.95, whilst ICC range for individual domains was respectively 0.87–0.97 [28] and 0.80–0.94 [29]. However, two studies presented domains that had a problem with the repeatability. Moen et al. validated the Norwegian version of the 36-item WHODAS 2.0 by conducting research amongst patients admitted to specialized somatic rehabilitation, including 455 people (47%) with diseases of the musculoskeletal system and connective tissue. The ICC was 0.87 for a total score, but for the DoDC was 0.63, which is slightly below the expected result [31]. Similarly, Garin et al. obtained a total score of 0.74, however for the DoGA domain ICC was only 0.2 [32].

The SEM associated with the total Polish version of 36-item WHODAS 2.0 was 2.34 in our study, and similarly Silva et al. found the SEM in Portuguese version amounts to 2.94 points [29]. Our study indicates that clinicians/researchers can take into account that the total Polish version of 36-item WHODAS 2.0 score falls within 2.34 points over a short time interval. We used the MDC to assess when true change had occurred in the individual patient’s Polish version of 36-item WHODAS 2.0 score. The MDC for the Total score was 6.49 points. Silva et al. obtained a slightly higher value of this parameter—8.15 points [29].

The floor and ceiling effect for the total score of the Polish version of 36-item WHODAS 2.0 was not present, with reference to the maximum acceptable level (15%) proposed by Terwee et al. [41]. However, floor effects, which have been reported in previous studies [18, 41–43, 46], were present in two of our domains—DoC (39.53%) and DoGA (18.60%). The study by Moen et al. demonstrated floor effect also in the same domains as our own study, and additionally with the highest percentage in the DoSC (53.7%) [31]. However, the study by Garin et al. indicated, that the floor effect was not relevant, but quite a high ceiling effect was present in almost all domains, especially for DoSC (53.6%) [32]. The occurrence of the floor effect in our study in the DoC may indicate a limited incidence of problems with communication skills and cognitive thinking in patients with OA, whilst insignificant problems in the DoGA prove limited impact of this disease on building relationships with other people. Kutlay et al. points out that the possible causes of the floor effect may be unsuitability of the 36-item WHODAS 2.0 to differentiate the least severe disability in these domains [28].

Fifteen a priori hypotheses were put forward to evaluate the construct validity of the Polish version of 36-item WHODAS 2.0. As hypothesized, scores that represent the same areas correlated strongly, indicating that both questionnaires measure a similar construct. At the same time, scores that represent less convergent regions correlate moderately. As a result of the analyses conducted, 12 out of 15 a priori hypotheses, i.e. 80%, were confirmed. According to Terwee et al. [41], this indicates a high construct validity of the questionnaire for the group of people with knee and hip OA, as less than 25% of the hypotheses were rejected. Baron M. et al. showed results convergent with ours that the 36-item WHODAS 2.0 total score was strongly correlated with the SF-36 2.0 physical component score (PCS) and moderately correlated with the SF-36 2.0 mental component score (MCS) [50]. Kutlay et al. also confirmed their established a priori correlations between the 36-item WHODAS 2.0 and the pain and physical sections of the WOMAC and psychosocial sections of the Nottingham health profile [28].

As hypothesized, the Polish version of 36-item WHODAS 2.0 questionnaire, demonstrate appropriate discriminatory power, i.e. significantly (*p* ≤ 0.001) differentiates between people with different functional status according to the WOMAC 3.1 and with different intensity of pain according to NRS (*p* ≤ 0.05). Similar results obtained by Posl et al. in which highly significant mean differences were found between groups (no-mild pain vs. strong pain) in people with musculoskeletal diseases for the following domains: DoGA, DoSC, DoLA, and DoP [30]. The study by Garin et al. showed that almost all the 36-item WHODAS 2.0 scores demonstrated statistically significant differences (*p* ≤ 0.001) between working patients and those not professionally active due to chronic illness [32]. Recent evidence has also indicated the appropriate discriminant power of the 36-item WHODAS 2.0 in patients with musculoskeletal disorders [29, 50–52].

A significant change in all domains of the Polish version of 36-item WHODAS 2.0 and in total score was found between test 1 and test 3 (7-week interval). All results decreased significantly, therefore a significant reduction in the degree of disability of the respondents was achieved as a result of an inpatient rehabilitation. ES and SRM were calculated to assess the responsiveness of the Polish version of 36-item WHODAS 2.0. Except DoC (which showed small responsiveness, ES = − 0.13, SRM = − 0.26), all other domains and Total score showed a moderate degree of responsiveness (ES: from − 0.32 to − 0.63, SMR: from − 0.48 to − 1.09). The lower degree of responsiveness in the field of DoC in our study can be explained by the presence of the floor effect, i.e. the absence of disability in this area in about 40% of the patients. Therefore, improvement in cognitive functions cannot be expected in this group (*N* = 51) as a result of rehabilitation. Posl et al. obtained moderate degree of responsiveness for domains: DoGA, DoLA and DoP, and small degree of responsiveness for the remaining domains [30]. Garin et al. analyzed the responsiveness of the 36-item WHODAS 2.0 with the use of ES, which for the domain range was from − 0.3 to − 0.7, so it was consistent with the results we obtained [32]. Meesters et al. also obtained a moderate degree of responsiveness (ES = − 0.34 and SRM = − 0.35) 6 weeks after discharge [49].

Federici et al. stressed that the 36-item WHODAS 2.0 is suitable for assessing health status and disability in a variety of settings and the most important issue for rehabilitation is that MCID score for the WHODAS 2.0 should be established [27]. As a result of analyses in our study, we found that the MCID for the Polish version of 36-item WHODAS 2.0 Total score in patient after rehabilitation for knee or hip OA is 3.29 ± 0.37. Patients experienced a significant change in their health in terms of mobility only when their MCID score changed by an average of 5.15 ± 0.69. However, they experienced a significant change in their health in terms of cognitive functions, when MCID score changed by an average of 0.89 ± 0.32. According to Shulman et al. the MCID is sensitive to different populations and clinical scenarios, so a range of MCID estimates may exist for a given PROMs depending on the context in which it is used [53]. However, a similar trend as in the present one in the evaluation of MCID for the 36-item WHODAS 2.0 questionnaire was observed by Ćwirlej-Sozańska et al. on a group of people with low back pain who also benefit from inpatient rehabilitation. The researchers obtained a score for MCID in case of the total WHODAS 2.0 score 4.87 ± 0.24. The largest MCID was demonstrated both in this study for DoM (7.93 ± 0.70), and the smallest also for DoC (1.71 ± 0.347). Therefore, similar trends are observed in the size of the MCID parameter for changes after rehabilitation amongst patients with various musculoskeletal problems [35].

### Limitations and strengths

The current study limitations is inclusion to the research of a small sample drawn from a single rehabilitation clinic. Samples of patients treated in different settings can be representative of the whole spectrum of limitations in functioning that patients with OA may have. Additionally, the inclusion of a sample containing more people at greater levels of disability could reduce the floor effect observed in DoC and DoGA in this study. As final note, there was a gender discrepancy in the sample group, about 75 percent of the group were female.

The benefits include the use of a standardized methods for evaluation of both reliability, validity and responsiveness of the Polish version of the 36-item WHODAS 2.0. A further bonus is the correlation between the results of our study and the results reported by other authors who have made linguistic adaptation and validation studies of the 36-item WHODAS. At the same time, it is the first study in Poland and one of several in the world to analyse the usefulness of the 36-item WHODAS 2.0 questionnaire in assessing the disability of OA patients.

### Future considerations

The lack of clarification of the factor structure of the questionnaire suggests that in future studies a larger the test group would be required [54]. However, the sample used in this study provided input into another, multi-site, pooled data study involving patients with various musculoskeletal problems that will clarify the factor structure of the Polish version of the 36-item WHODAS 2.0 through the use of CFA. Furthermore, by involving patients with various health problems and/or undergoing various medical interventions in the study, it will be possible to continue the analysis in the field of MCID assessment.

## Conclusions

The Polish version of the 36-item WHODAS 2.0 is a reliable and valid questionnaire for assessing disability based on the ICF model which can be applied to patients with hip or knee OA. It can also accurately capture changes in disability after rehabilitation in these groups of patients. Therefore, the Polish version of the 36-item WHODAS 2.0 constitutes a considerable support in clinical practice and in National and International scientific research projects, relating to patients with hip or knee OA. Further studies using 36-item WHODAS 2.0 as an outcome measure are required in the rehabilitation research field.

## Data Availability

The datasets used and/or analyzed during the current study are available from the corresponding author on reasonable request.
